# Sources of variation in the 3dMDface and Vectra H1 3D facial imaging systems

**DOI:** 10.1038/s41598-020-61333-3

**Published:** 2020-03-10

**Authors:** Julie D. White, Alejandra Ortega-Castrillon, Ciara Virgo, Karlijne Indencleef, Hanne Hoskens, Mark D. Shriver, Peter Claes

**Affiliations:** 1 Department of Anthropology, The Pennsylvania State University, University Park, PA United States; 2 Department of Electrical Engineering, ESAT/PSI, KU Leuven, Leuven, Belgium; 3 Medical Imaging Research Center, UZ Leuven, Leuven, Belgium; 4 Department of Human Genetics, University Hospitals Leuven, Leuven, Belgium; 5 Murdoch Childrens Research Institute, Melbourne, Victoria Australia; 6 Department of Biomedical Engineering, University of Oxford, Oxford, United Kingdom

**Keywords:** 3-D reconstruction, Hardware and infrastructure, Image processing, Quality control

## Abstract

As technology advances and collaborations grow, our ability to finely quantify and explore morphological variation in 3D structures can enable important discoveries and insights into clinical, evolutionary, and genetic questions. However, it is critical to explore and understand the relative contribution of potential sources of error to the structures under study. In this study, we isolated the level of error in 3D facial images attributable to four sources, using the 3dMDface and Vectra H1 camera systems. When the two camera systems are used separately to image human participants, this analysis finds an upper bound of error potentially introduced by the use of the 3dMDface or Vectra H1 camera systems, in conjunction with the MeshMonk registration toolbox, at 0.44 mm and 0.40 mm, respectively. For studies using both camera systems, this upper bound increases to 0.85 mm, on average, and there are systematic differences in the representation of the eyelids, nostrils, and mouth by the two camera systems. Our results highlight the need for careful assessment of potential sources of error in 3D images, both in terms of magnitude and position, especially when dealing with very small measurements or performing many tests.

## Introduction

With its ease of use and portability, 3D imaging technology has transformed clinical diagnostic methods and research into the causes and consequences of morphological variation. 3D imaging systems have quick capture speeds, are minimally invasive, and provide researchers and clinicians with the ability to create detailed, comprehensive, and realistic images for use in assessing variation or planning treatments.

Variation in human facial structure across individuals is patently visible, but difficult to comprehensively quantify given the complex and multipartite three-dimensional nature of the face. The technological shift to using 3D images, first with sparse sets of a few dozen or less manually-indicated landmarks and now with automated dense configurations of thousands of landmarks, has greatly enhanced our understanding of clinical, genetic, and evolutionary aspects of facial variation. In a clinical setting, geometric morphometrics on 3D images have improved our understanding of the facial form and variability associated with dysmorphic disorders like Down syndrome^1^ and allowed clinicians to compare facial shape before and after surgery^2^. In academic research, geometric morphometric analysis on sparsely-placed landmarks has helped facilitate a better understanding of the trends in facial evolution^3^ and morphological relationships among hominin species^4^. Recent advancements in the dense registration of facial images have improved our understanding of dysmorphic facial morphologies^5–7^, the relationships between genes and facial features^8–11^, and the evolutionary processes shaping global variation in nose shape^12^.

Historically, 3D images have been compared and standardized using landmarks placed by human operators. These landmarks are placed on anatomically significant regions, depending on the research question or clinical purpose, and intra- and inter-observer variability among the landmarks must be assessed prior to analysis^13–16^. However, variation among landmarks resulting from researcher placement is only one source of potential variation. All imaging systems have some technical error, an inherent level of variation introduced as a function of the hardware and software used by the camera, which cannot be reduced by the operator. In studies using a single imaging system on all participants, this technical error is standardized across all participants. However, in studies using multiple imaging systems, variability due to camera system could introduce systematic bias. Lastly, especially pertinent to systems that take sequential images, like the Vectra H1, or laser scanners that must be moved around the object, like the Konica Minolta Vivid 900, participant movement between captures additionally introduces variation. Participants are generally asked to remain as still as possible and gaze forward with a neutral expression^17^, but micromovements around the eyes and lips are often made without conscious thought, and previous assessments of variation in repeated measurements of sparse and dense landmarks have indicated greater variation around the mouth and eyes relative to the rest of the face^18–21^.

In this study, we did not aim to measure the accuracy of either imaging system, or the extent to which a 3D construct made by the imaging system truthfully represents the object in reality. Though this is an important and relevant topic, the accuracy of both imaging systems has already been the purpose of several prior studies, with the common conclusion that the individual systems produce results that very closely match dimensions measured directly on the object via traditional anthropometry^19,22–24^. Instead, given the advancements in registration technology, expanding our analyses from a few dozen landmarks to several thousand, and the increase in opportunities for combined analyses of data collected with different imaging systems, we sought to explore: (1) the presence and extent of random artifactual variation originating from registering images using the MeshMonk non-rigid surface registration toolbox^25^ as opposed to traditional human-placed landmarks; (2) the presence and extent of non-random variation as a result of technical error from the camera and participant movement during imaging; and (3) potential systematic biases in the manner in which images are photographed and represented by two different, commonly-used, camera systems: the 3dMDface and Vectra H1.

**Figure 1:**
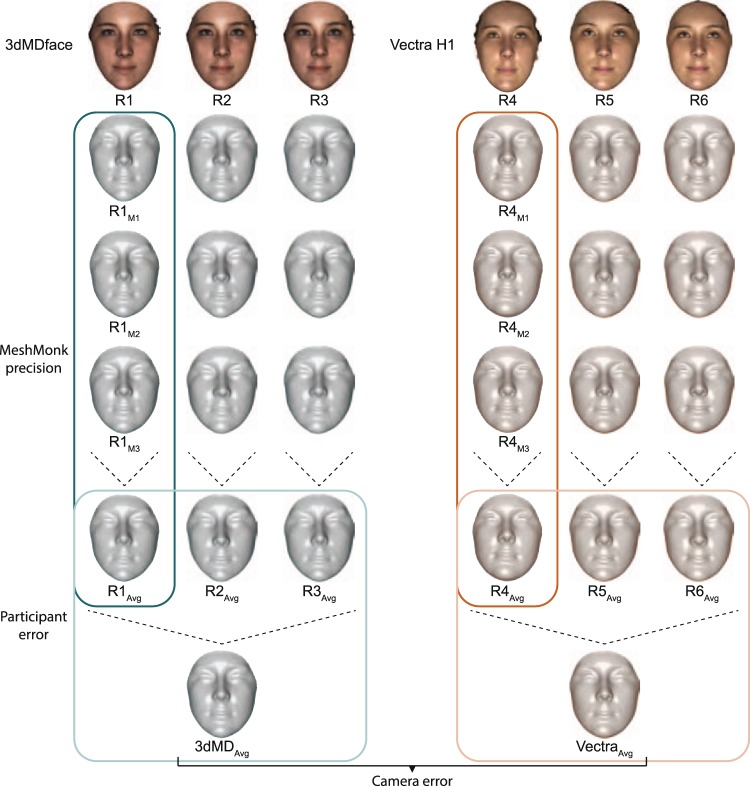
Study design. Three images of each participant were taken with the 3dMDface (left) and the Vectra H1 (right), for a total of 6 image replicates per participant. Each image was then registered three times with MeshMonk, resulting in a total of 18 quasi-landmark configurations per individual. Comparisons of each set of registrations to the average of all three registration iterations (column-wise) led to estimates of MeshMonk precision. Within-camera comparisons of the three average registrations to the overall average for that camera gave estimates of participant error per camera as well as technical error per camera, when analyzing the mannequin images. Lastly, the average quasi-landmark configuration from each camera was compared to create estimates of error due to imaging system. Individual imaged is one of the authors.

Following Aldridge, *et al*., we define precision as the difference between repeated measures of the same image^26^, and assessed the precision of the MeshMonk registration process by comparing quasi-landmark placement across three registrations of the same image (‘MeshMonk precision’; Fig. 1). Quasi-landmarks are not strictly anatomical, like many landmarks placed manually by human observers, but are constructed such that the vertices are positioned relative to their relationship with surrounding vertices and the position of vertices on one individual are comparable to the position of those vertices on another individual (i.e. they are homologous). Variation due to human movement between image captures (‘participant error’), was assessed by comparing sequential images of the same person (Fig. 1). Variation introduced by the camera machinery and software was calculated by comparing sequential images of a mannequin head (‘technical error’). Lastly, form variability resulting from systematic differences between cameras was assessed by comparing the same individuals imaged with two different camera systems (‘camera error’; Fig. 1).

## Results

**Figure 2:**
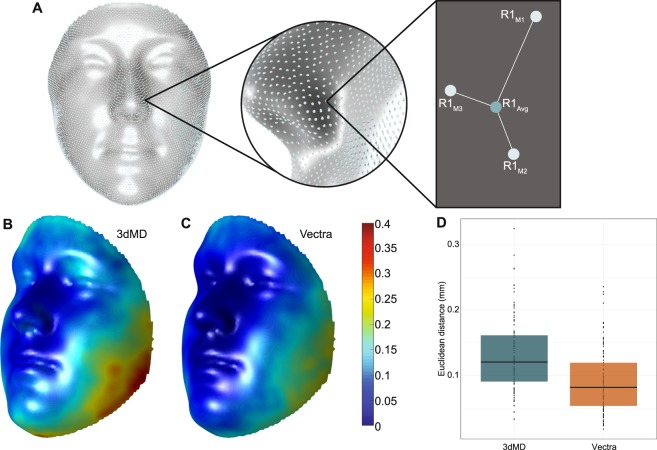
MeshMonk precision for participants. (**A**) For each replicate image of each individual, precision was calculated by first averaging together the three registration iterations (e.g. R1_Avg_), then calculating the distance from each iteration (e.g. R1_M1_, R1_M2_, R1_M3_) to the average. Replicate “1” is used as the example in this figure. The three distances were then averaged and this process was repeated across all 7,160 quasi-landmarks. **(B)** Precision (mm) for the 3dMDface images, averaged across all replicate images of all participants (*n* = 105). **(C)** Precision (mm) for the Vectra H1 images, averaged across all replicate images of all participants (*n* = 105). Scale bar in mm applicable to both images. **(D)** Precision per image, stratified by camera.

The precision of the MeshMonk registration process was calculated as the average Euclidean distance for each quasi-landmark between each MeshMonk registration iteration (e.g. R1_M1_, R1_M2_, and R1_M3_) and the average of all three registration iterations (e.g. R1_Avg_), averaged across all quasi-landmarks and all replicates. This value was 0.13 mm (*SD* = 0.07 mm, *min* = 0.02 mm, *max* = 0.37 mm) for the 3dMDface and 0.09 mm (*SD* = 0.06 mm, *min* = 0.01 mm, *max* = 0.26 mm) for the Vectra H1, with the Vectra H1 values, on average, lower than the 3dMDface values (Figs. 2, S2). The MeshMonk precision estimate averaged across all three replicate mannequin images was 0.1 mm (*SD* = 0.09 mm, *min* = 0 mm, *max* = 0.45 mm) for the 3dMDface and 0.02 mm (*SD* = 0.04, *min* = 0 mm, *max* = 0.26 mm) for the Vectra H1 (Fig. S3). When comparing the precision values for 3dMDface and Vectra H1 across mannequin replicates, the Vectra H1 replicate images all had lower mean values and tighter distributions, compared to the 3dMDface replicates (Fig. S3).

**Figure 3:**
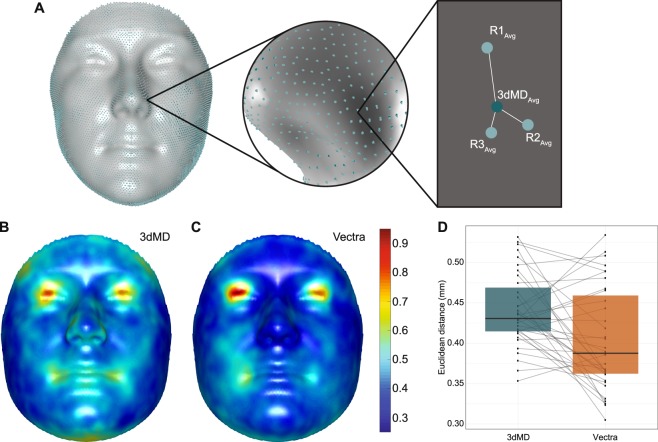
Participant error. (**A**) To calculate the error from participant movement between images, the three registration iterations for each replicate image were averaged (e.g. R1_Avg_, R2_Avg_, R3_Avg_) and aligned using a non-scaled, non-reflected GPA. The average quasi-landmark configuration for each person on the camera was calculated by averaging together the three replicate images (e.g. 3dMD_Avg_). The participant error was estimated by calculating the distance from each replicate image to the average quasi-landmark configuration. The three distances were then averaged and this process was repeated across all 7,160 quasi-landmarks. **(B)** Participant error (mm) for the 3dMDface images, averaged across all participants (*n* = 35). **(C)** Participant error (mm) for the Vectra H1 images, averaged across all participants (*n* = 35). Scale bar in mm applicable to both images. **(D)** Participant error plotted per camera. Each point represents the average across all quasi-landmarks for one individual, with the grey lines connecting that individual’s 3dMDface and Vectra H1 values.

The error due to participant movement was calculated as the Euclidean distance between replicate images of the same person on the same camera and that person’s overall average image from the camera. Each replicate image was represented as the average of all three MeshMonk registrations of the image (e.g. R1_Avg_, R2_Avg_, and R3_Avg_) and the person’s overall average image from the camera was represented as the average of these replicates (e.g. 3dMD_Avg_). The participant error, averaged across all landmarks and individuals was 0.44 mm (*SD* = 0.07 mm, *min* = 0.31 mm, *max* = 0.82 mm) for the 3dMDface quasi-landmarks and 0.40 mm (*SD* = 0.06 mm, *min* = 0.29, *max* = 0.92 mm) for the Vectra H1 quasi-landmarks (Figs. 3, S4). For the mannequin, the technical error averaged across the three 3dMDface replicates was 0.35 mm (*SD* = 0.14 mm, *min* = 0.06 mm, *max* = 1.34 mm), and 0.34 mm (*SD* = 0.13 mm, *min* = 0.05 mm, *max* = 0.87 mm) averaged across the Vectra H1 replicates, and the distributions of error were all very similar (Fig. S5).

**Figure 4:**
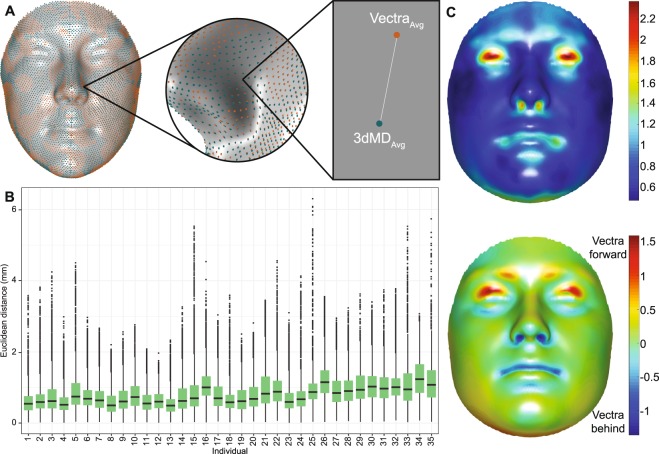
Camera error for participants. (**A**) To calculate the camera error for each person, all quasi-landmark configurations for each camera were averaged (3dMD_Avg_ and Vectra_Avg_) and aligned using a non-scaled, non-reflected GPA. The camera error was estimated by calculating the distance between the average 3dMDface configuration and the average Vectra H1 configuration. This process was repeated across all 7,160 quasi-landmarks. **(B)** Boxplots of camera error for all participants. **(C)** *Top*: Distribution of camera error across the face, calculated by averaging the Euclidean distance values (mm) per quasi-landmark over the 35 participants. *Bottom*: Distribution of displacement along the normal vectors across the face, going from the 3dMDface image to the Vectra H1 image, after GPA alignment. Red values are those where the direction of the vector is positive, indicating that the Vectra H1 image is more outwardly displaced relative to the 3dMDface image. Blue values are those where the direction of the vector is negative, indicating that the Vectra H1 image is inwardly displaced or recessed relative to the 3dMDface image. Values have been averaged across all 35 participants and are unit-less.

When comparing the 3dMDface and Vectra H1 systems, we found that the average Euclidean distance was 0.85 mm (*SD* = 0.23 mm, *min* = 0.48 mm, *max* = 2.37 mm) for the participant dense quasi-landmark configurations (Fig. 4) and the average Euclidean distance between the mannequin 3dMDface and Vectra H1 images was 0.70 mm (*SD* = 0.28, *min* = 0.06 mm, *max* = 2.48 mm; Fig. S6). These comparisons were performed by calculating the Euclidean distance at each landmark between the average image of each person from the two camera systems (e.g. 3dMD_Avg_ and Vectra_Avg_).

In the repeated measures ANOVA, camera was a significant predictor of variation in both participants (*p* = 0.02; Table 1) and mannequin dense quasi-landmark configuration (*p* = 0.01; Table S1), after considering the impact of individual variation, image replicate (Camera:Individual interaction), and MeshMonk registration (Camera:Individual:Replicate interaction). In contrast, when considering only 19 traditional landmarks, camera was not a significant predictor of form variation (*p* = 0.37; Table 2) after considering the effects of individual and image replicate.

**Table 1:** ANOVA on participant dense quasi-landmark configurations.

Covariate	Df	SS	MS	Rsq	F	Pr (>F)^100^
Camera	1	13688	13688	0.000141	1.0399	0.02
Individual	34	16191932	476233	0.166900	36.1777	0.09
Camera:Individual	34	447567	13164	0.004613	1.7627	0.01
Camera:Individual:Replicate	140	1045493	7468	0.010777	17.1218	0.01
Residuals	420	183187	436	0.001888		
Total	629	97015652				

After non-scaled, non-reflected GPA alignment, an ANOVA was used to assess the relative contribution to form variation in the participant dense quasi-landmark configurations. Type III sums of squares was used, with 100 iterations and formula of y ~ Camera:Individual:Replicate. Camera was treated as a fixed effect and individual and replicate as random effects.

**Table 2:** ANOVA on 19 traditional landmarks.

Covariate	Df	SS	MS	Rsq	F	Pr (>F)^100^
Camera	1	10	9.68	0.00024	0.5959	0.37
Individual	34	19657	578.16	0.47947	35.4204	0.01
Camera:Individual	34	555	16.32	0.01354	2.9581	0.01
Residuals	140	773	5.52	0.01884		
Total	209	40998				

After non-scaled, non-reflected GPA alignment, an ANOVA was used to assess the relative contribution to form variation in the 19 traditional landmarks automatically indicated on the participants. Type III sums of squares was used, with the 100 iterations and formula of y ~ Camera:Individual. Camera was treated as a fixed effect and individual as a random effect.

## Discussion

In this study, we report variation in the 3dMDface and Vectra H1 imaging systems at multiple levels using densely-registered images. The precision of quasi-landmark placement by the MeshMonk registration toolbox was quite high, with error values estimated from participant images averaging 0.13 mm (*SD* = 0.07 mm) for the 3dMDface and 0.09 mm (*SD* = 0.06 mm) for the Vectra H1. When visualized on the registration template, these errors were localized to the lower sides of the face (Fig. 2B,C), which is indicative of the fidelity with which MeshMonk is able to find anatomical correspondence in areas of the face usually of greater interest to biologists (e.g. eyes, nose, and mouth). This localization of errors to the sides of the face could be due to the cleaning process implemented to remove hair, clothes, and ears. Since cleaning is implemented in each registration iteration, each time the set of points along the outer edge of the face is likely to differ slightly, leading to slightly different final registration results.

Also of interest is the higher error values produced when the toolbox was used to register 3dMDface images compared to Vectra H1 images (Fig. 2D). We hypothesize that this result is because the Vectra H1 produced images with large point clouds, containing about 90 K points, while the 3dMDface produced images with smaller average point clouds (~35 K points), and higher density point clouds should allow for a more precise localization of points on the target surface that correspond to each of the template vertices.

Unless explicitly scripted, each MeshMonk registration iteration starts from scratch each time, with the user placing five positioning landmarks and the toolbox searching for correspondences between the template and the target vertices, so each registration iteration results in slightly different correspondence definitions, likely additionally explaining the presence of slight random artifactual variation evidenced by this analysis. For these reasons, researchers seeking to eliminate this small variation while using the MeshMonk registration toolbox can use the average registration of multiple MeshMonk iterations as the basis of their analysis, as we have done when assessing participant and technical error. Lastly, this study focused on the precision of the MeshMonk registration toolbox on individuals and a mannequin that display typical-range facial morphology, meaning that wide divergences between template and target are unlikely. It is possible that, given a target that differs widely from the template used, such as individuals with extreme facial dysmorphology, the precision of the MeshMonk toolbox would decrease. The extent to which this occurs and the algorithmic parameters needed to reproducibly represent images exhibiting extreme morphology is one of our areas of active research.

Despite best intentions and instructions to remain as still as possible, live human participants are never truly motionless. When standing freely and without some sort of stabilizing force, some natural involuntary movement, referred to as sway, occurs, because the human body must produce small muscle bursts in order to stay balanced and standing^27^. Though the impact of sway on surface imaging has traditionally been considered negligible, significant differences were found when comparing the biometric recognition performance of 3D laser scanning images with participants either standing freely or sitting with their head stabilized against a wall^28^. Additional movement can occur when the participant makes microexpressions, which are typically defined as brief (200–500 ms) spontaneous facial expressions that appear when a person suppresses or conceals an emotion^29^, for example smiles or laughter. In our experience, these can occur when the operator and participant are communicating around the time of image capture and when the Vectra H1 operator moves directly in front of the participant and unconsciously makes eye contact. Lastly, involuntary movements in the eyes are especially common, though not always related to microexpressions. Saccade movements, the rapid ballistic movements of the eyes that abruptly change the point of fixation, for example to read a book or gaze around a room, can occur involuntarily even when the eyes are fixed on a target^30^. Thus, even though we asked participants to gaze at a fixed point on the wall, it is likely that the eyes of many participants darted away from that point during the imaging process.

In this study, we quantified the variation in facial images across three sequential images of the same participant. It is worth noting that this ‘participant error’ value is composed both of variation due to human movement and variation due to the machine imaging process, or ‘technical error’. In this study, the average participant error values were 0.44 mm (*SD* = 0.07 mm) for the 3dMDface images and 0.40 mm (*SD* = 0.06 mm) Vectra H1 images. These values are similar to previous reports of mean participant error (0.41 mm) found when comparing differences in the length of pairwise linear distances calculated from a set of 61 surface landmarks placed on two individuals and captured using the 3dMDface system 20 times^31^. As expected, these errors are localized around the eyes and mouth (Fig. 3B,C), likely due to the participant moving their eyes to blink or track the operator and mouth movements from microexpressions or talking in-between imaging captures. When assessing the distribution of 3dMDface and Vectra H1 values averaged for each participant, the Vectra H1 has a lower mean value, but a much wider distribution than the 3dMDface images. This could be a result of the three composite images required by the Vectra H1, as opposed to the single shot required by the 3dMDface, as the Vectra H1 participants must remain still for longer and the magnitude of sway that each participant exhibits will vary. Another relevant consideration is that participants are generally asked to sit down when taking their 3dMDface photo, but not when taking their Vectra H1 photos, as a sitting participant would make the Vectra H1 image capture unwieldy. Thus, the wider range of participant error values for the Vectra H1 could be explained by a greater variation in participant’s ability to stabilize themselves while standing.

As the above participant error values was composed of both error due to participant movement and the machine imaging process, we used a mannequin head to determine the amount of error attributable to internal variation in the imaging machinery and software of each camera system. For the 3dMDface, 3dMD reports this error at <0.2 mm root mean square error or better^32^. Canfield does not report the technical error for the Vectra H1 on their website, however, Tzou, *et al*. report the technical error value for the Vectra H1 as “>0.1 mm (x,y,z)” in their Table 1, though they do not describe how this value was obtained^33^. In this study, the average technical error is 0.35 mm (*SD* = 0.14 mm) for the 3dMDface images and 0.34 mm (*SD* = 0.13 mm) for the Vectra H1 images and is randomly distributed across the face (Fig. S5), indicating that there are no systematic biases in the location of technical errors for either imaging system. Taking this into account, we can re-evaluate the average participant error in the densely landmarked images that is introduced by participant movement as, on average, 0.09 mm for the 3dMDface and 0.06 mm for the Vectra H1 (calculated by subtracting participant error and technical error). However, it is important to remember that these are averages across many quasi-landmarks, and the magnitude of error due to participant movement is much higher around the eyes and mouth (Fig. 3; *max*_*3dMD*_ = 0.82 mm around the eyes, *max*_*Vectra*_ = 0.92 mm around the eyes), a pattern not seen in the distribution of technical error on the mannequin face (Fig. S5), meaning that there is a predictable pattern to the locations most affected by participant movement and researchers should take care when studying variation in the shape of these regions.

The levels of participant and technical error reported in this study, using images gathered under optimal lighting conditions, following manufacturer guidelines, and with adult participants who carefully followed instructions, are likely lower than those expected if images were collected under suboptimal conditions. For this reason, researchers should endeavor to control for factors that are likely to increase extraneous variation in images by standardizing participant body and head positioning, the distance from the participants to the camera, and lighting conditions, and should incorporate analyses of landmark precision in their study designs whenever possible, and especially when working with hardware or software for the first time or in a new context. Researchers with the opportunity to do so can also reduce some of the participant error by taking multiple images of the same individual and averaging them together, as we have done in the calculations of camera error.

Lastly, when comparing the 3dMDface and Vectra H1 camera systems, we find that there are systematic biases in the location and direction of error values between the two cameras. On average, the Euclidean distance between the participant dense quasi-landmark configuration was 0.85 mm (*SD* = 0.23 mm), with the maximal values located around the eyelids, nostrils and oral fissure (Fig. 4C, *top*). The mannequin images additionally show similar error values, with the average being 0.70 mm (*SD* = 0.28 mm) across all quasi-landmarks. The distribution of camera errors across the mannequin images are more randomly distributed across the face, though slightly higher values are present around the nostrils and corners of the mouth (Fig. S6A). Most importantly, using the normal displacement vectors, we find that the Vectra H1 on average produces an image, relative to the 3dMDface image, that is more outwardly displaced around the eyelashes and more inwardly displaced around the inner nostrils and oral fissure (Fig. 4C, *bottom*). The nostril depression is replicated on the mannequin images, though the mouth and eye results are not (Fig. S6B), likely because the mannequin we used did not have eyelashes or a very deep oral fissure. Though our results cannot further clarify the impact of point cloud density on camera error, we suspect that the difference in density, and thus image resolution, could explain overall biases in the 3D images produced and that systematic processing differences are additionally complicit in the production of consistent differences between the 3dMDface and the Vectra H1 around the eyes, nose, and mouth.

From their analyses of traditional sparse landmarks, two recent reports by Camison *et al*.^18^ and Liberton *et al*.^21^ report that the 3dMDface and Vectra H1 are highly comparable and could be combined in most situations. In an ANOVA on the aligned images, we found that camera was a significant predictor of variation in landmark position for the dense quasi-landmark configurations (*p*_100_ = 0.02; Table 1), but not when only considering 19 landmarks (*p*_100_ = 0.37; Table 2). This could explain why the Camison *et al*. and Liberton *et al*., analyses, which both focused primarily on sparse landmarks, did not find meaningful differences. Taken together, these results indicate that there are systematic differences in the landmark configurations produced by the two systems, but these may only be discernible when studying dense landmarks. However, even studies using sparse landmarks should be aware of the potential for biases, as the facial locations most affected by differences in camera system (the eyes, nose, and mouth) are often of most interest to biological researchers and the site of placed landmarks. For these studies, or any using multiple camera systems, we suggest that researchers investigate the possibility of false results in their analyses stemming from camera differences and control for these differences by meta-analyzing or including camera system as a covariate.

This study thoroughly explores four potential sources of variation in 3D facial images using two camera systems. On average, between 0.09 mm (Vectra H1) and 0.13 mm (3dMDface) error can be attributed to the use of the MeshMonk registration toolbox. In this study, the average amount of error attributable to the internal mechanics of the camera systems used (i.e. the technical error) was 0.34 mm (Vectra H1) and 0.35 mm (3dMDface). Accounting for this technical error, participant movement adds less than ~0.1 mm additional variation, with the total average difference between sequential images of the same person being 0.40 mm (Vectra H1) and 0.44 mm (3dMDface). For studies using both the Vectra H1 and 3dMDface camera systems, 0.85 mm average error can be expected due to differences in the camera systems. Though focused on faces, with this study we highlight the need to carefully consider sources of error in studies using geometric morphometric methods, regardless of structure, that is especially relevant as advancements in dense registration technology now allow us to better quantify 3D shapes and databases of 3D images continue to grow.

## Methods

### Participant recruitment

In this study, 35 volunteer adult participants were recruited and imaged three times using both the 3dMDface and the Vectra H1 cameras. Most participants took the 3dMDface and Vectra H1 photos within seven days of each other. Participants were asked to pull any hair out of their face, remove all jewelry from the facial area, keep a closed mouth, and maintain a neutral facial expression, following standard image acquisition protocols^17^. All data collection and experimental protocols were approved by and performed in accordance with the relevant guidelines and regulations from the Ethics Committee Research UZ/KU Leuven institutional committee (protocol #S56392), and all participants signed a written informed consent form before participation. To additionally assess error without the possibility of participant movement, a mannequin head was imaged, registered, and analyzed in the same manner as the human participants (Fig. S1). The mannequin surface was dusted with white talcum powder to reduce glare from the shiny finish on the mannequin head.

### Image capture

The 3dMDface (3dMD, Atlanta, GA) and Vectra H1 (Canfield, Parsippany, NJ) are two currently available systems for 3D imaging, with the 3dMDface being very common and extensively tested^22–24,26,33–35^. The 3dMDface is a stationary rig composed of either 2 or 3 cameras positioned at angles that provide overlapping views of the face from different angles. The overlapping images are captured at the same time and immediately stitched together by 3dMDface software into the 3D structure. The time needed to take the participant’s image and for that image to be stitched together by the 3dMD software is approximately 2 minutes, allowing very short participant interactions that make this camera useful when imaging small children or other persons who may not be able to remain immobile for longer durations^20^. The 3-camera 3dMDface system used for these analyses was stationed in a room with overhead light and no windows, thereby reducing the chance of extraneous light patterning affecting the image. After calibration following the manufacturer’s instructions, each participant was seated and positioned symmetrically between the left and right camera pods, with the position of the chair unchanged between image captures or participants. During image capture, our participants were instructed to tilt their heads slightly upward and keep a neutral facial expression with their mouths closed and their eyes open and gazing directly forward. For the current study design, three complete 3dMDface images were taken of each participant without participant adjustment in-between images.

The Vectra H1 is a more recent portable handheld SLR-type camera that requires the operator to take multiple photos from different angles to ensure full facial representation. The Vectra H1 images were taken in a different room than the one containing the 3dMDface camera, though still windowless and with overhead light. Following the manufacturer guidelines, three sequential photos of each participant were taken that focused on the middle of the participant’s left cheek, the upper lip, and the middle of the participant’s right cheek, with the distance of the camera to the subject calibrated using built-in ranging lights. In the first and last images, the camera is held approximately 45° to the left and right of the participant, at chest level, and angled upward. In the middle image, the camera is held at face level and pointed directly at the philtrum. During the entire image acquisition process, in which three complete Vectra H1 image sets were taken, the participants were instructed to remain standing as still as possible and gaze forward with a neutral expression and their mouths closed. Allowing for operator adjustment between the three individual photos, a single 3D image capture process takes about 30 seconds^21^. If the camera is tethered to a computer with Canfield imaging software, the entire imaging plus 3D stitching takes about five minutes, though operators also have the option to store images on the camera’s memory card and process them at a later time, thereby reducing the participant time taken.

### Landmark registration

Each replicate participant image was registered three times using the MeshMonk registration toolbox, which resulted in 7,160 homologous quasi-landmarks covering the entire facial surface per facial image^25^. This study design generated a total of 3 registrations × 3 images × 2 cameras = 18 dense quasi-landmark configurations per person (*n* = 630 total configurations; Fig. 1). The registration process is more thoroughly detailed in White *et al*.,^25^. Briefly, each 3D surface image is cleaned of ears, hair, clothing, and extraneous 3D artifacts so that only the facial surface remains. Five positioning landmarks are then roughly placed on the image (outer corners of the eyes and lips, and tip of the nose). The algorithm proceeds with a scaled rigid alignment based on the iterative closest point algorithm^36^, in which the position, orientation, and scale of the template is changed to better fit the shape of the target. Subsequently, a non-rigid registration is performed that more finely alters the shape of the template to match the shape of the target surface. During this step, a visco-elastic model is enforced, ensuring that points that are close to one another move coherently^37^. The two-step nature of this process, in which the template is first roughly aligned, and then more closely molded to fit the shape of the target, is robust to variation in the positioning of the five initial landmarks, though slight variations in their position could explain differences in multiple registrations of the same image.

To compare the relative contribution of errors found using dense landmark configurations with those from sparse landmark configurations, we automatically placed 19 anatomically-relevant landmarks on each of the replicate images using a coordinate conversion process, resulting in 3 images × 2 cameras = 6 sparse landmark configurations per person. These landmarks are the 19 validation landmarks presented in White *et al*.,^25^, which also more thoroughly details the method with which they are automatically placed. None of the participants present in the current study were analyzed in our previous work.

### Precision and error calculations

#### MeshMonk precision

Since the MeshMonk surface registration toolbox is initialized using five manually-placed landmarks and the definition of correspondences between template and target is novel in each iteration, the registration process can produce slightly different results each time. Previous iterations of the MeshMonk registration toolbox were reported to have a quasi-landmark precision of 0.2 mm^38^, with this result attributed to an algorithmic parameter in the registration process (Chapter 3 of Claes, 2007^39^). In this study, we have updated these statistics for the current version of the MeshMonk toolbox by calculating the average Euclidean distance between three registrations of the same image (e.g. R1_M1_, R1_M2_, R1_M3_) and their average quasi-landmark configuration (e.g. R1_Avg_). Because the coordinate space does not change between the surface registration iterations, we performed this analysis without superimposition.

#### Participant and technical error

Micromovements from the participant can cause additional variation in form. To assess this participant-level error, the average quasi-landmark configuration from the three registrations was calculated for each replicate image of the same individual from each camera (e.g. R1_Avg_, R2_Avg_, R3_Avg_; *n* = 210 total configurations). Separate non-scaled and non-reflected Generalized Procrustes Alignments (GPA) were used to align the three replicate images from each camera system, ensuring that the replicate images from a single individual within each camera system were aligned to each other, not to the images of any other individuals or with images of the same individual from the other camera. We then calculated the Euclidean distance between each replicate and the average of the three replicates (e.g. 3dMD_Avg_). The above process was performed on the mannequin images to assess the technical error within each camera system.

#### Camera error

To assess variation due to camera system, we used the average of the three replicates for each individual on each camera system to provide one 3dMDface and one Vectra H1 image for each person (e.g. 3dMD_Avg_ and Vectra_Avg_; *n* = 70 total configurations). The two images were aligned using a non-scaled and non-reflected GPA per individual, then the Euclidean distance between the aligned images was calculated. To provide additional insight into the directionality of the differences between the 3dMDface and the Vectra H1, the displacement along the normal vectors (normal displacement) going from 3dMDface to Vectra H1 was calculated.

### Relative contributions to landmark variation

The experimental design employed is a complete randomized block design with repeated measures, thus the magnitude of variance within groups is attributable to differences made by the observer on the same object, or in our case the differences made by the MeshMonk toolbox in registering the same image multiple times (MeshMonk precision). This design also allows us to partition the magnitude of variance attributable to replicate images of the same person (participant error), and that attributable to the camera system (camera error). For these calculations we used a repeated measures ANOVA on the non-scaled and non-reflected GPA-aligned coordinates, with camera as a fixed effect and individual and replicate as random effects^40–42^. For the set of 19 traditional landmarks, an ANOVA using the non-scaled and non-reflected GPA-aligned coordinates was similarly performed, with camera as a fixed effect and individual as a random effect.

## Supplementary information

Supplemental Material.

## Data Availability

The informed consent with which the data were collected does not allow for dissemination of identifiable data to persons not listed as researchers on the IRB protocol. Thus, the full surface 3D facial images used for validation cannot be made publicly available. In the interest of reproducibility, we have provided the raw images and quasi-landmark configurations for the mannequin as well as the participant coordinates of the 19 automatic landmarks used in the analysis of relative contributions to sparse landmark variation. We have also included the R code containing all analyses. These data are available in the following GitHub repository: https://github.com/juliedwhite/Vectra_vs_3dMD. The MeshMonk code and tutorials are available at https://github.com/TheWebMonks/meshmonk.
