# Supplementary material for: Sources of variation in the 3dMDface and Vectra H1 3D facial imaging systems
Source: Sci Rep. 2020 Mar 10;10:4443. doi: 10.1038/s41598-020-61333-3 (PMC7064576; doi:10.1038/s41598-020-61333-3)
Supplement: Supplementary file 1 — Supplemental Material. [file 41598_2020_61333_MOESM1_ESM.docx]

Supplemental Information

**Sources of variation in the 3dMDface and Vectra H1 3D facial imaging systems**

Julie D. White^1^, Alejandra Ortega-Castrillon^2,3^, Ciara Virgo^1^, Karlijne Indencleef^2,3^, Hanne Hoskens^2,4^, Mark D. Shriver^1^, Peter Claes^2,3,4,5,6^

Supplemental Figures


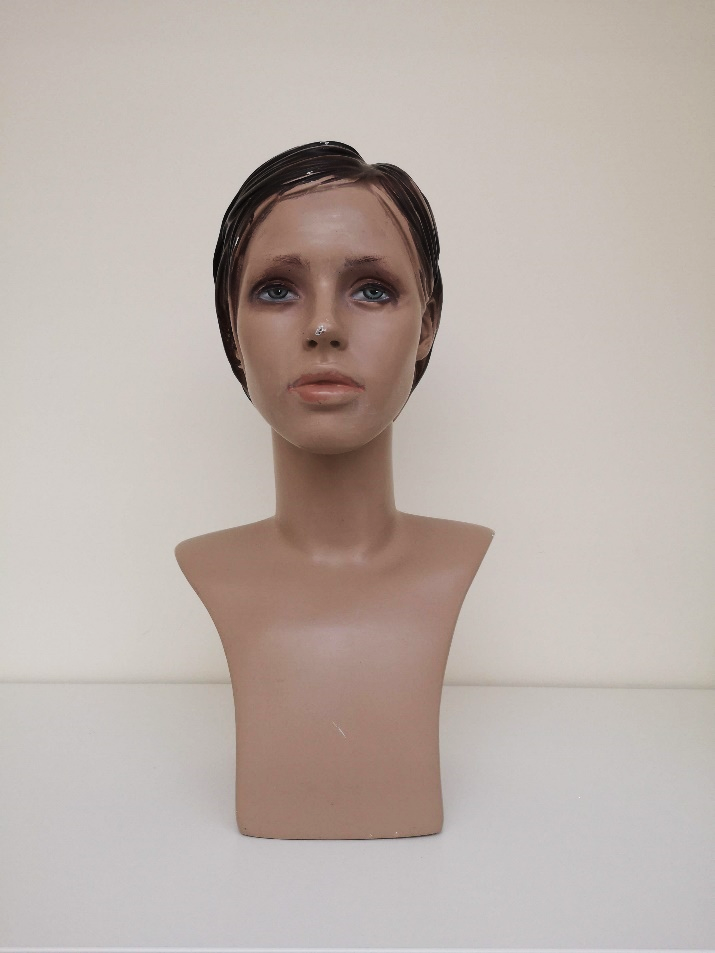

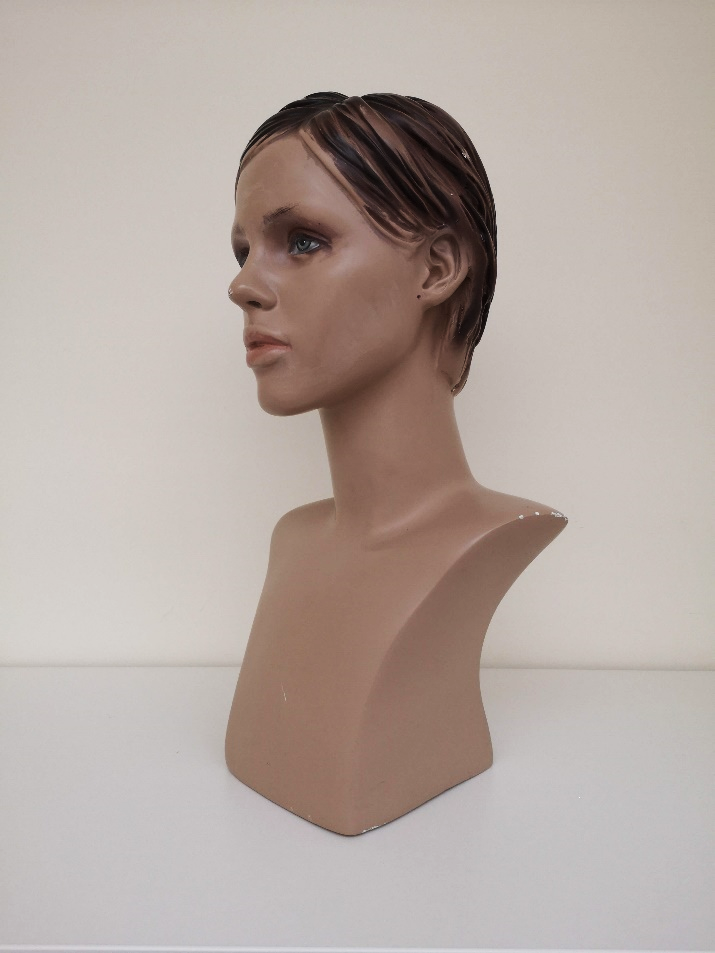


**Figure S1. Mannequin head.** Image above is the mannequin head used in this analysis, which is for sale in the Netherlands (<https://www.etalagepoppengigant.nl/nl/presentatie-hoofd-hv-g>). Because of the shiny facial surface, the mannequin head was dusted with talcum powder prior to imaging.


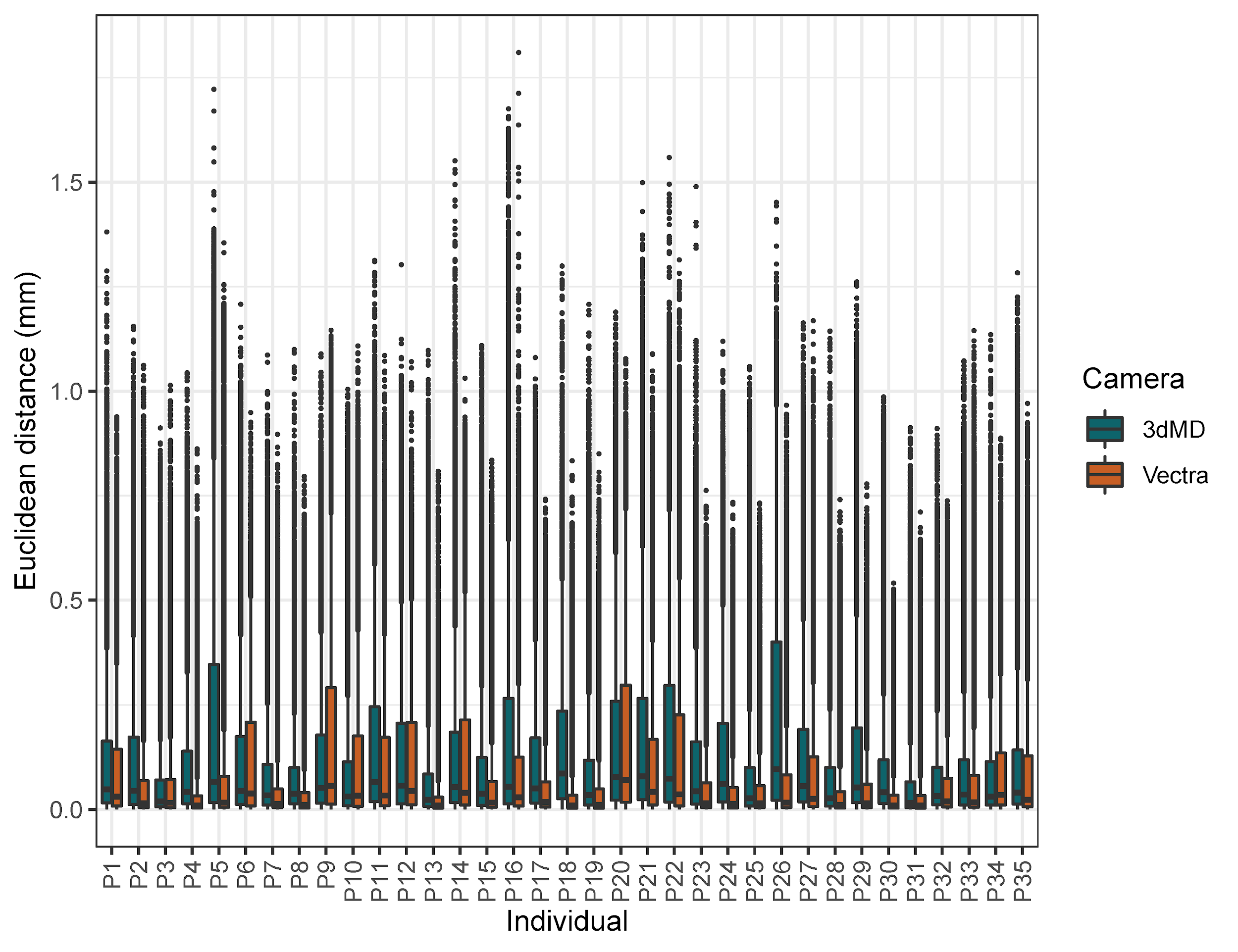


**Figure S2. MeshMonk precision per person.** MeshMonk precision values for each landmark plotted per person, stratified by camera. Values are average Euclidean distance (mm) between each registration iteration (e.g. R1_M1_, R1_M2_, R1_M3_) and the average of the three registrations for that individual (e.g. R1_Avg_). Values are plotted per person, with each boxplot containing the values from all three replicate images of that person on each camera. Calculation of the precision values are the same as depicted in Figure 2A.


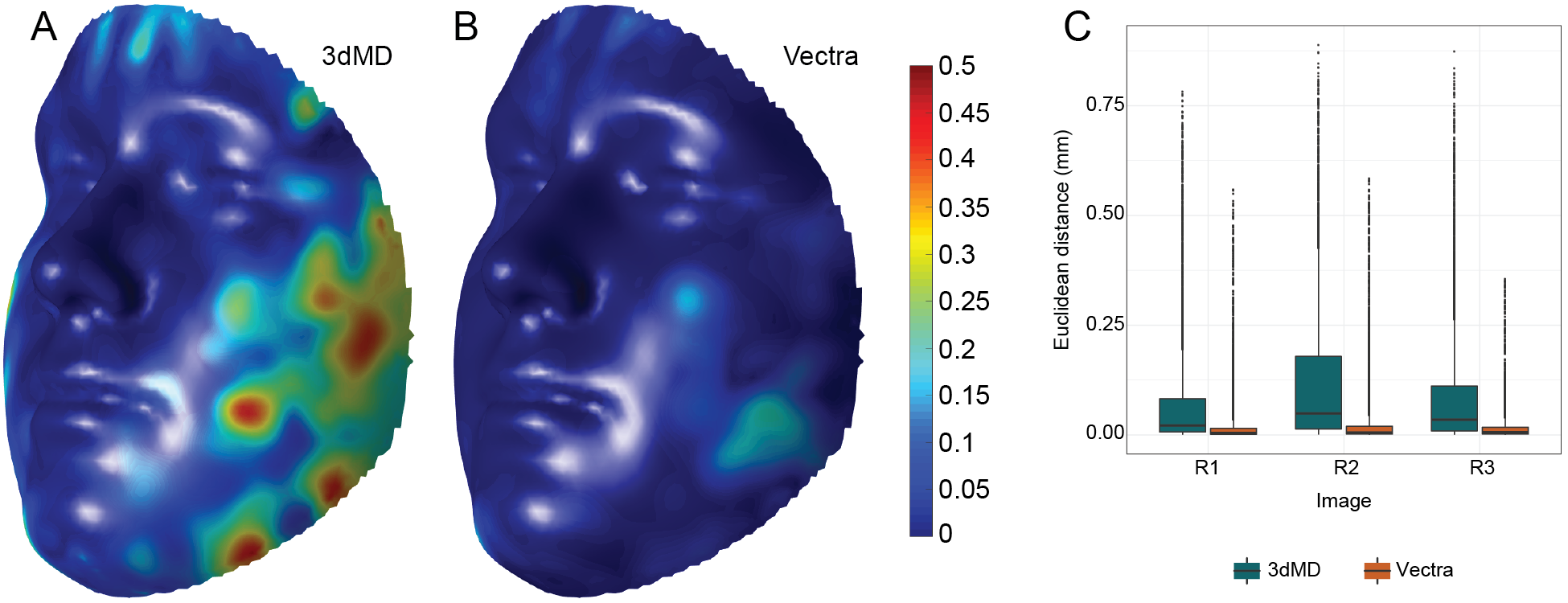


**Figure S3. MeshMonk precision for mannequin.** (A) The MeshMonk precision values (mm) for the 3dMDface, averaged across all three replicate images of the mannequin. (B) MeshMonk precision values (mm) for the Vectra H1, averaged across all the three replicate images of the mannequin. Scale on the right applies to both facial images. (C) MeshMonk precision values (mm) per replicate image, stratified by camera. Calculation of the precision values are the same as depicted in Figure 2A.


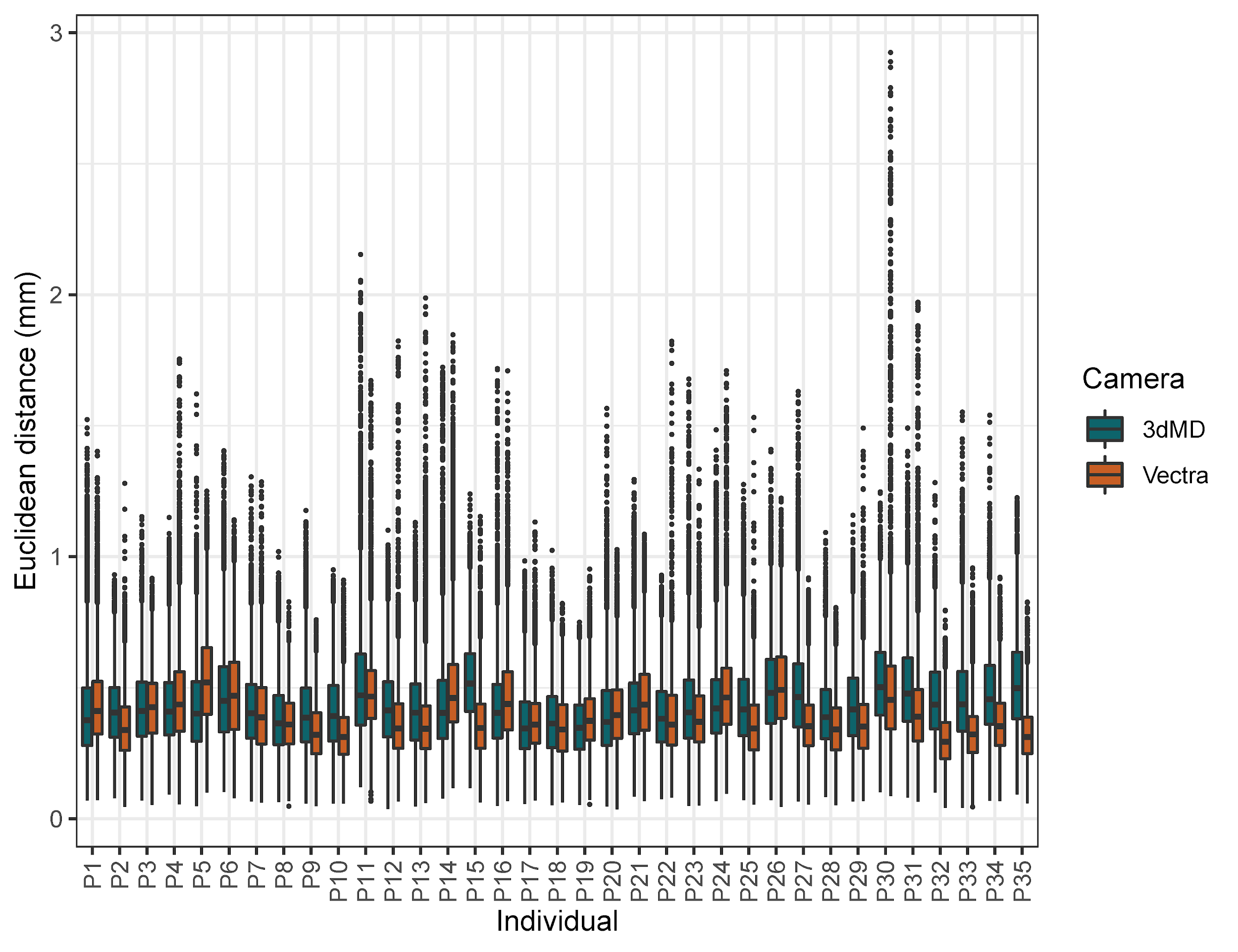


**Figure S4. Quasi-landmark participant error.** Participant error values for the dense quasi-landmark configurations plotted per person, stratified by camera. Values are the average Euclidean distance (mm) between each replicate image (e.g. R1_Avg_, R2_Avg_, R3_Avg_) and the average of the three replicate images for that individual (e.g. 3dMD_Avg_). Calculation of the error values is the same as that depicted in Figure 3A.


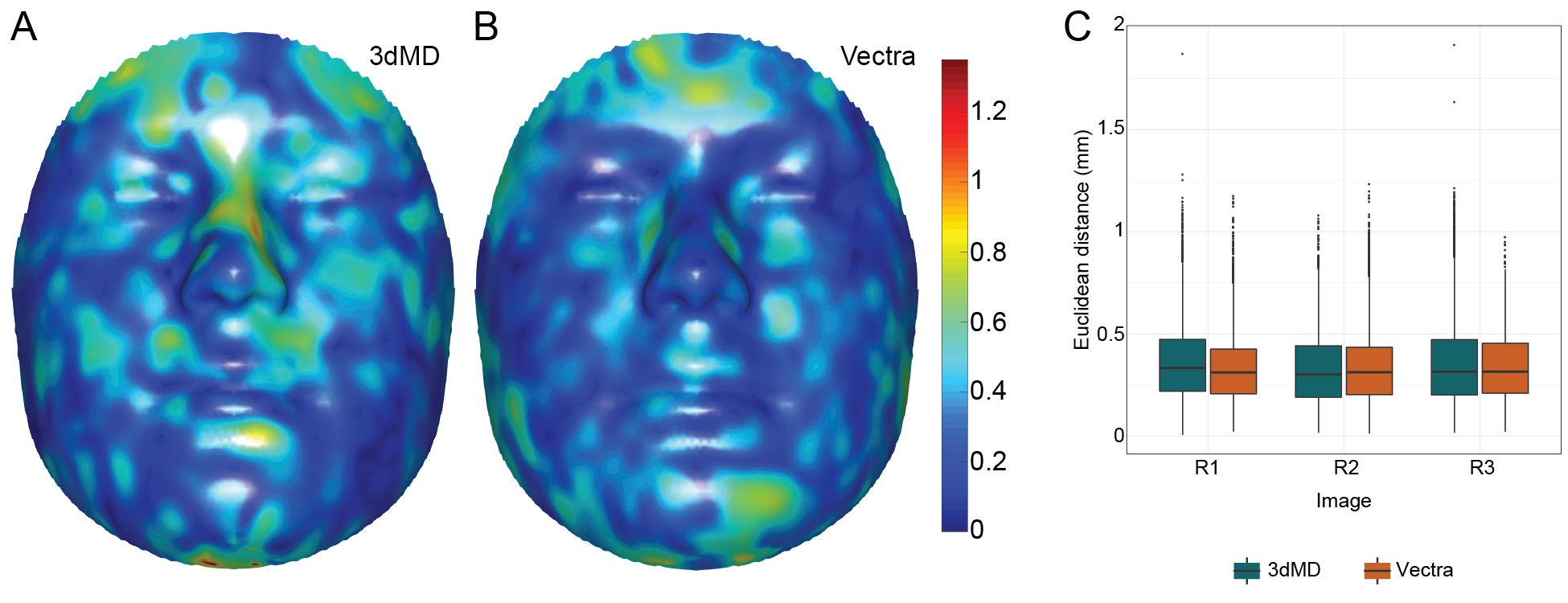


**Figure S5. Technical error using the mannequin images.** (A) The technical error (mm) for the 3dMDface mannequin images, averaged across the three replicate images. (B) Technical error (mm) for the Vectra H1 mannequin images, averaged across the three replicate images. Scale on the right applies to both images. (C) Technical error per replicate image, stratified by camera. Calculation of the error values is the same as that depicted in Figure 3A.


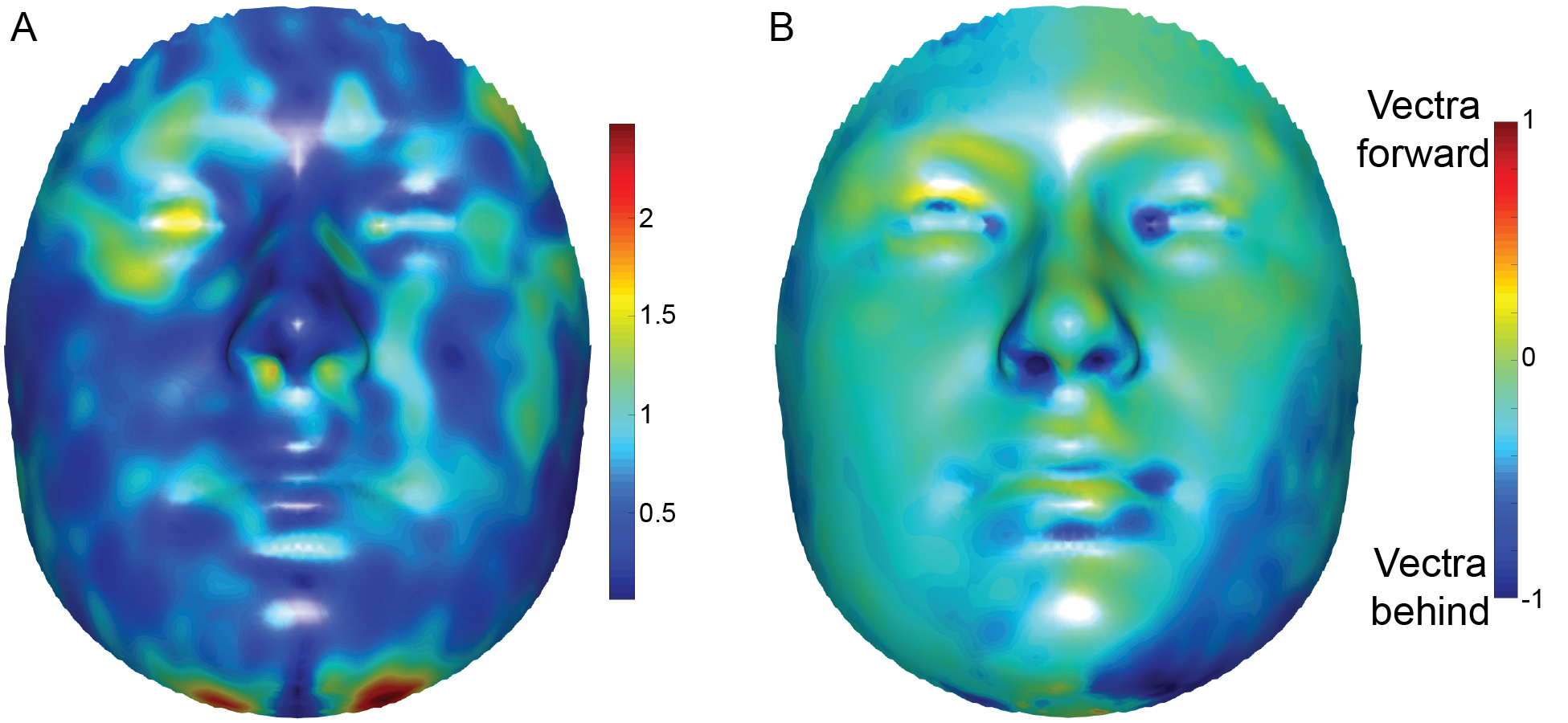


**Figure S6. Quasi-landmark camera error for the mannequin images.** (A) Distribution of Euclidean distance (mm) values across the face. (B) Distribution of displacement along the normal vectors across the face. Yellow and red values are those where the direction of the vector is positive, indicating that the Vectra H1 image is more outwardly displaced relative to the 3dMDface image. Blue values are those where the direction of the vector is negative, indicating that the Vectra H1 image is more inwardly displaced or recessed relative to the 3dMDface image.

Supplemental Tables

**Table S1. ANOVA on mannequin quasi-landmark shape configurations.** After non-scaled, non-reflected GPA alignment, an ANOVA was used to assess the relative contribution to shape variation in the mannequin images using Type III sums of squares and y ~ Camera:Replicate as the formula, with 100 iterations.

|  | **Df** | **SS** | **MS** | **Rsq** | **F** | **Pr (>F)^100^** |
| --- | --- | --- | --- | --- | --- | --- |
| **Camera** | 1 | 11040 | 11039.7 | 0.27667 | 63.166 | 0.01 |
| **Replicate** | 4 | 19495 | 4873.8 | 0.48857 | 27.887 | 0.01 |
| **Residuals** | 12 | 2097 | 174.8 | 0.05256 |  |  |
| **Total** | 17 | 39903 |  |  |  |  |
